# Predicting persistent back pain causing severe interference with daily activities among community-dwelling older adults: the OPAL cohort study

**DOI:** 10.1186/s12877-024-05504-1

**Published:** 2024-11-14

**Authors:** Esther Williamson, Maria T. Sanchez-Santos, Jeremy Fairbank, Lianne Wood, Sarah E. Lamb

**Affiliations:** 1 Nuffield Department of Rheumatology, Orthopaedics and Musculoskeletal Sciences, University of Oxford, Oxford, UK; 2 Faculty of Health and Life Sciences, University of Exeter, Exeter, UK

**Keywords:** Older adults, Back pain, Leg pain, Neurogenic claudication, Disability

## Abstract

**Background:**

Many older adults experience disabling back and leg pain. This study aimed to identify factors associated with back pain causing severe interference with daily activities over 2 years.

**Methods:**

Participants were 2,109 community-dwelling adults (aged 65–100 years; mean age 74.2 (SD 6.3)) enrolled in a prospective cohort study who reported back pain at baseline and provided back pain data at 2 years follow-up. Baseline data included demographics, socio-economic factors, back pain presentation and age-associated adverse health states (e.g. frailty, falls, walking confidence). At 2 years follow-up, we asked if they were currently experiencing back pain and if so, asked participants to rate how much their back pain interfered with their daily activities on a scale of 0–10. Severe back pain interference was defined by a rating of 7 or more. The association between baseline factors and severe back pain interference at two years was assessed using logistic regression models.

**Results:**

At two years, 77% of participants (1,611/2,109) still reported back pain, 25% (544/2,083) also reported leg pain and 14% (227/1,611) reported severe back pain interference with activities. Improvements in symptoms were observed over the two years follow-up in 880/2,109 participants (41.7%), 41.2% (869/2,109) of participants report no change and worsening symptoms was reported by 17.1% (360/2109) of participants. After adjusting for back pain troublesomeness at baseline, factors associated with reporting severe interference were adequacy of income (careful with money [OR 1.91; 95% CI 1.19–3.06]; prefer not to say [OR 2.22; 95% CI 1.11–4.43]), low endorsement of exercise in later life (OR 1.18; 95% CI 1.02–1.37), neurogenic claudication symptoms (OR 1.68 (95% CI 1.15–2.46)], multisite pain (OR 1.13; 95% CI 1.02–1.24) and low walking confidence (OR 1.15; 95% CI 1.08–1.22).

**Conclusion:**

After adjusting for baseline pain severity, we identified five factors that were associated with severe pain limitation at two years follow-up among a cohort of community dwelling older people reporting back and leg pain. These included other pain characteristics, walking confidence and attitude to activity in later life. We also identified a socioeconomic factor (perceived adequacy of income). Future research should focus on whether identifying individuals using these risk factors in order to intervene improves back pain outcomes for older people.

**Supplementary Information:**

The online version contains supplementary material available at 10.1186/s12877-024-05504-1.

## Introduction

Back pain (BP) and associated leg symptoms are common in older people [1]. Qualitative studies suggest that living with persistent, restricting BP has the potential to impair activities of daily living, disrupt sleep and exercise participation, lead to sadness, irritability and worsening health, and feelings of isolation [2]. We have demonstrated in a cross-sectional analysis that back and leg pain are associated with age-associated adverse health states including falls, frailty and mobility decline and reduced quality of life with the largest impact in people with back and leg pain with a neurogenic claudication pattern [1]. For many older people, back and leg symptoms are persistent. In a cohort of older people presenting to primary care for treatment for their BP, 20% had suffered with back symptoms for 5 years or more [3]. At two years follow up, only 17% of this cohort no longer reported BP or back pain related disability [4]. Older people will experience BP alongside age-related changes to the musculoskeletal system including sarcopenia (age-related muscle loss), osteoarthritis (changes to articular cartilage) and osteoporosis (loss of bone density) [5]. These changes contribute to structural changes within the spine (for example, increased kyphosis) that can result in loss of spinal sagittal alignment predisposing older people to reduced standing balance and falls [6]. Many older people will also experience adverse health states associated with older age (sometimes called geriatric syndromes) such as frailty, falls, immobility, incontinence, cognitive impairment and sleep disturbance [7]. These age-related health states are associated with poorer health outcomes [8] but their associations with BP outcomes was unclear. When seeking to understand what leads to persistent and disabling BP in older people, we need to consider the broader picture of ageing taking into account these age associated adverse health states alongside our understanding of chronic pain based on the biopsychosocial model of pain [9]. The biopsychosocial model approach to pain conceptualises pain as being a multidimensional interaction between physical (physiological), psychological, and social factors which contribute to an individual’s experience of pain [9].

There are several cohorts studying risk factors for persistent disabling or restrictive BP in older people with follow up ranging from 3 months [10], 12 months [11, 12] and 2 years [4, 13]. These studies have studied many factors consistent with the biopsychosocial model of pain and known to play a role in persistent pain, and have identified risk factors that are not specific to older people including higher intensity pain or greater disability at baseline, older age, being female, more comorbidities and psychological factors (pain catastrophizing, depression, low recovery expectations). Less attention has been paid to potential age-related risk factors that may contribute to the biopsychosocial model of BP when applying this model to older people. Van den Berg included radiological parameters related to spinal degeneration and found multilevel osteophytes were associated with poor outcomes at 12 months in a cohort of 543 older adults [14]. Also related to degenerative changes, a diagnosis of spinal stenosis has also been associated with poor outcomes at 12 months in a cohort of 5220 participants [12]. Falls were also studied in this cohort [12]. A history of falling in the past 3 weeks was associated with poor outcome at 1 year follow up but it was no longer associated at 2 years follow up [4, 12]. Makris et al. included variables pertinent to ageing including physical capacity (measured by the Short Physical Performance Battery, grip strength and lower limb weakness) and cognitive impairment in their cohort study of 731 participants followed up for 126 months [15], of which none were associated with BP outcomes.

Using data from a large cohort of community dwelling older adults, the aims of this study are (1) to estimate the proportion of this cohort who report persistent back and leg pain over a 2-year period and (2) to identify baseline risk factors (including common age-related adverse health states) that are associated with the report of BP that causes severe interference with participants’ ability to undertake daily activities at 2-year follow up. We focus on pain resulting in substantial limitation of daily activities as the loss of ability to perform everyday tasks threatens an older person’s independence and puts them at risk of requiring care [16, 17].

## Methods

### Study design and participants

**Fig. 1:**
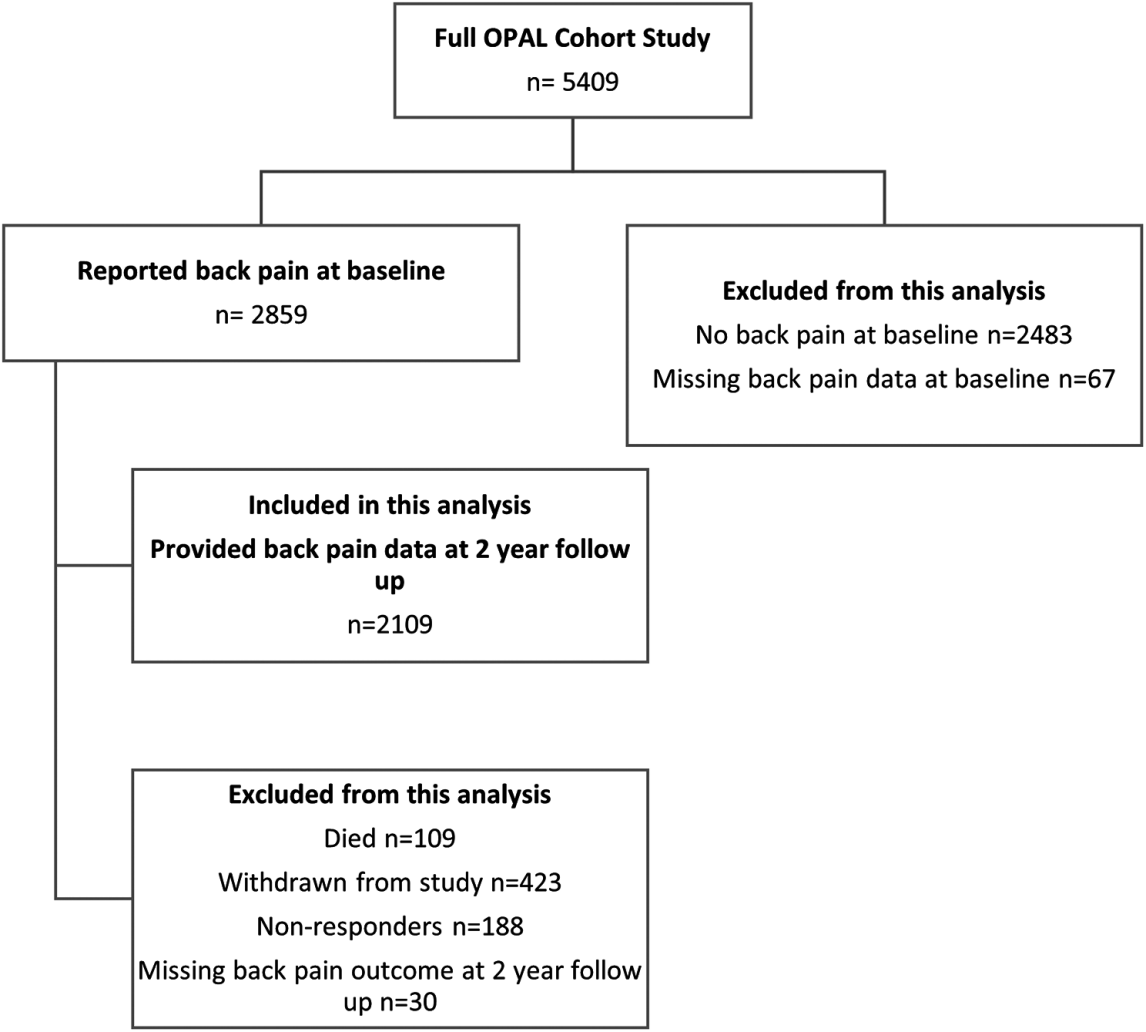
Study flowchart

The Oxford Pain, Activity and Lifestyle (OPAL) cohort study is a prospective cohort study of community dwelling older adults in England, UK. A full description of the cohort is published elsewhere [18]. We recruited 5,409 community dwelling older adults via 35 general practices in England. Participants were 65 years of age and older. For this study, participants who reported BP at baseline and completed the BP outcome question on the two-year follow up questionnaire were included (*N* = 2,109) (see Fig. 1). We compared the characteristics of individuals from the original OPAL cohort sample (*N* = 5,409) with those included in this study (*N* = 2,109) to understand if those who were included this study differed significantly from the overall cohort in case this was a potential source of bias.

### Data collection and definition of variables

#### Dependent variable (outcome)

The outcome for this analysis is the report of severe pain interference due to BP. At 2 years follow up, participant rated how much their back pain interfered with their daily activities (0 = no interference, 10 = unable to carry out the activities). This question was based on the Von Korff Pain Scale [19]. Severely interfering BP was defined as a report of ≥ 7/10. This cut-point has been used in cohorts of patient with pain to indicate severe pain interference [20, 21]. If a participant was no longer reporting back pain at 2 years follow up, then they were allocated a score of 0/10.

#### Independent variables (baseline factors)

##### Demographics

Demographic factors included age, sex, education and socioeconomic status.

Socioeconomic status was determined by:

Education: level of education was reported by participants.

Physical demands of occupation: participants rated the physical demands of their main occupation during their life as very light/light, moderate and strenuous/very strenuous.

Deprivation: participants were allocated an Index of multiple deprivation score (IMD (0-100 score)s based on their postcode [22] with a higher score indicating greater deprivation. IMD were divided into quintiles from least to most deprived in England.

Adequacy of income: we also collected the participant’s perception about adequacy of their income (quite comfortably off, able to manage without much difficulty, need to be careful with money; find it a strain to get by, prefer not to say) [23]. We combined “careful with money” and “find it a strain to get by” into one category.

#### General health

Body Mass Index (BMI): calculated using self-reported height and weight.

Comorbidities: participants indicated if their doctor or nurse had told them that they had any of the following health conditions: arthritis, angina or heart troubles, cancer, chronic lung disease, diabetes, digestive problems, high blood pressure, osteoporosis, Parkinson’s disease, peripheral vascular disease and stroke. The total number of comorbidities was created.

Anxiety and depression: measured using a single item from the Eq. 5D-5 L [24].

#### Lifestyle

Smoking: participants were classified as ex/current smokers or never smoked [25].

Physical activity: the amount of time spent being active each day was measured using a single question from the Rapid Assessment Disuse Index [26].

Attitude to exercise: We measured attitudes to exercise using a single question from physical changes subscale of the Attitudes to Ageing Questionnaire which assessed agreement with the statement: I keep fit and active as possible by exercising [27].

Function: Baseline ability to perform their usual activities was measured using ability to perform usual activities question from the Eq. 5D-5 L [24].

#### Pain

Report of back and leg pain: participants were asked if they were troubled by BP or related symptoms.

If ‘yes’, the participant was asked about:

Frequency: participants indicated how often they experienced symptoms (every day, most days, some days, few days, rarely).

Troublesomeness: participants scored how much they were troubled by their back pain (scored 1–5: extremely, very, moderately, slightly, not at all) [28].

Spread of symptoms: participant indicated whether symptoms had spread into the legs over the last 6 weeks (including questions to identify neurogenic claudication (NC)).

This information was collected at baseline and year two of follow up.

Back and leg pain categories: back and leg pain presentation was categorised into three mutually exclusive groups: (1) BP only; (2) BP and NC leg pain; (3) BP and leg pain that is not NC (non-NC). NC was defined as the presence of BP or other symptoms that travel from the back into the buttocks or legs and was worse when standing and/or walking and better when sitting and/or bending [29]. Using this definition, participants reporting leg pain made worse by standing or walking and made better with sitting or bending were classified as having leg pain likely to be NC [29].

Multisite pain: We measured the presence of multi-site pain over the last 6 weeks using an adapted version of the Nordic Pain Questionnaire [30, 31]. Participants reported if they have experienced pain in six different body sites (neck, shoulders, elbows, hands/wrist, hips, knees, feet/ankles).

#### Age-related adverse health states

Frailty: The Tilburg Frailty Indicator was competed and scored out of 15 [32]. A score of ≥ 5 identifies an individual as frail [32].

Mobility decline: assessed using a 5-point scale constructed for the study asking “Compared to one year ago, how would you rate your walking in general?” Participants reporting worsening of walking was classified as having mobility decline.

Walking self-efficacy: participants rated their confidence to walk half a mile using a question from the Modified Gait Self-efficacy Scale [33].

Falls: Falls in the last year were collected using Prevention of Falls Network Europe recommendations by asking, “In the last 12 months, have you had any fall including a slip or trip following which you have come to rest on the ground, floor or lower level [34]? .

Incontinence: Incontinence was reported using the urinary incontinence item from the Barthel Index [35, 36]. Participants reported frequency of urinary incontinence (never, less than once per week, less than once per day, more often or uses a catheter). Participants who selected never or less than once per week were considered continent.

Sleep: Participants rated their sleep quality (very good, fairly good, fairly bad or very bad) during the past month using the sleep quality overall rating from Pittsburgh Sleep Quality Index [37]. Participants who reported fairly bad or very bad sleep quality were classified as ‘poor sleep quality’.

Grip strength: Reduced muscle strength was measured using the self-report of problems in their daily life due to lack of strength in their hands from the Tilburg Frailty Indicator.

We also collected this data at 2 years follow up.

#### Analysis

We summarised back pain presentation at 2-year follow-up and presented the baseline variables stratified by presence of severe pain interference at two years. Absolute change in BP troublesomeness from baseline to follow up stratified by back and leg pain presentation was calculated and described.

Missing data on independent baseline variables varied, with the least for age, sex, IMD, number of comorbidities and multisite pain (0 missing) and most for BMI (*n* = 76, 3.6%) (see supplementary materials - Table S1). In total, 217/2,109 (10.2%) of eligible participants had missing data on ≥ 1 independent factors. Multiple imputation by chained equations (MICE) was used to address potential bias and increase precision as a result of missing data. MICE assumes that data are Missing At Random (MAR). All the independent factors together with the outcome variable in the imputation model were included. Twenty multiple datasets were generated, and the resulting estimates were combined using Rubin’s rules. Further details of the multiple imputation process are described in supplementary data. All variables with missingness were imputed before predictive models were generated.

Univariable and multivariable association between independent variables and the outcome at two years was examined using logistic regression models. Odds Ratio (OR) and 95% confidence intervals (95%CI) were calculated.

Three sequential models were constructed: firstly, we included the independent variables of demographic, general health and lifestyle factors (Model 1); then we added pain-related factors (Model 2); and finally, age-associated adverse health factors at baseline were added (Model 3). As baseline severity is consistently associated with persistent pain, all models were adjusted for baseline BP troublesomeness. Only factors associated with the outcome (*p* < 0.05) were considered candidates to enter in the next step of the analysis to build the final model. All the analyses were performed by using Stata 17.0.

### Ethical approval

The London - Brent Research Ethics Committee (16/LO/0348) approved this study on the 10th of March 2016.

## Results

At baseline, 2,859/5,409 (52.9%) OPAL participants reported experiencing BP. Of these participants, 2,109/2,859 (73.8%) returned the two-year follow up questionnaire and completed at least some of the BP variables. These participants were included in this analysis (Fig. 1).

Seventy-seven percent of participants (1,611/2,109) were still reporting BP at two years (Table 1) with half reporting BP only (1,050/2,083). Leg pain was also reported by 25% of respondents (544/2,083) with 15% (332/2083) reporting leg pain in a neurogenic claudication pattern and 10% (212/2,083) reporting non-neurogenic claudication like pain. Of those still reporting BP (with or without leg pain) at two years follow up, 376/1,608 (23.4%) reported their pain was not at all or slightly troublesome, 441/1,608 (27.4%) reported their pain was moderately troublesome and 791/1,608 (49.2%) reported it to be very or extremely troublesome. 14% (227/1,611) reported that their back pain caused severe interference with their daily activities. The group most commonly reporting severe pain interference were those who reported BP and NC leg pain.

We compared the baseline characteristics of those included in these analyses with the entire OPAL cohort at baseline. We found that included individuals had slightly higher rate of age-related adverse health factors but were similar results in all other characteristics suggesting there was no selection bias in this study (See Table S2).

**Table 1:** Report of back pain at two years follow up

Variables measured at 2 years follow up	Total	Report of severe interference with daily activities	
		No	Yes
**Report of back pain (*n* = 2**,**100)**			
No	489 (22.9%)	489 (100%)	-
Yes	1,611 (77.1%)	1,367 (85.8%)	227 (14.2%)
**Back pain presentation (*n* = 2**,**083)**			
No back pain	489 (23.2%)	489 (100%)	-
Back pain only	1,050 (49.8%)	965 (91.9%)	85 (8.1%)
BP + non-NC leg pain	212 (10.1%)	174 (82.0%)	38 (18.0%)
BP + NC leg pain	332 (15.7%)	228 (68.7%)	104 (31.3%)

**Fig. 2:**
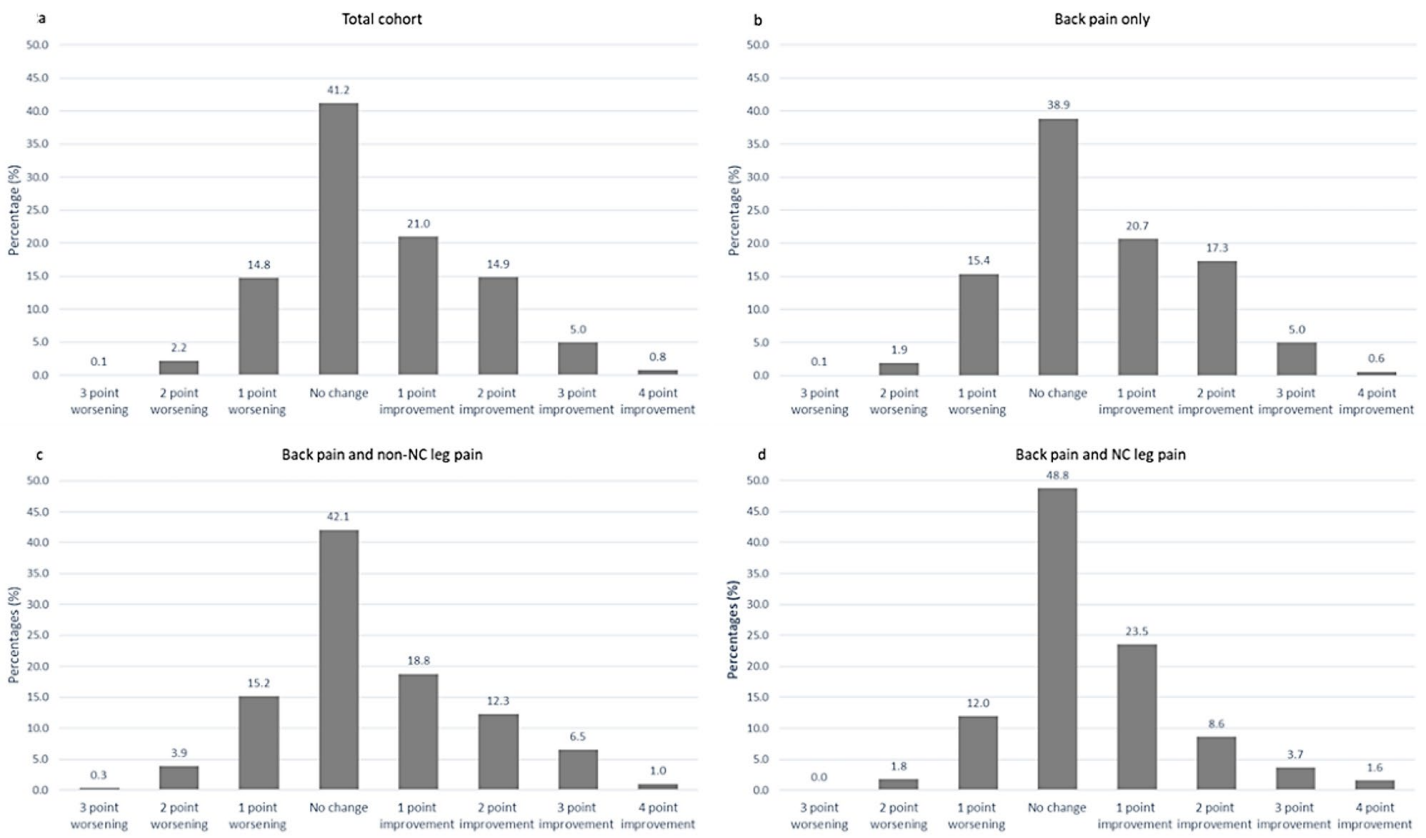
**a**-**d** Absolute change in back pain troublesomeness between baseline and two year follow up stratified by baseline back pain presentation

Improvements in BP troublesomeness were observed over the two year follow up in 880/2,109 participants (41.7%) including those who no longer reported pain (Fig. 2). Improvement was most often reported by those reporting BP only at baseline. No change in BP troublesomeness was reported by 41.2% (869/2,109) of participants and was most common among those reporting back and NC leg pain at baseline. Worsening symptoms was reported by 17.1% (360/2109) and most often reported by participants with back and non-NC leg pain.

Baseline variables stratified by pain interference at 2 years are presented in Table 2.

**Table 2:** Baseline variables stratified by presence of severe pain interference at two years

**Baseline factors**		Overall **(N = 2,109)**	severely interfering back pain (≥7) at 2 year follow up	
			No (n = 1,876)	Yes (n = 233)
**Demographic**				
Age (years), mean (SD)		74.2 (6.3)	74.1 (6.2)	75.2 (6.8)
Sex, n (%)	Female	1,170 (55.5)	1,023 (54.5)	147 (63.1)
Education, n (%)	Higher education	807 (38.3)	736 (39.2)	71 (30.5)
	Secondary	1,152 (54.6)	1,021 (54.4)	131 (56.2)
	None or primary	138 (6.5)	109 (5.8)	29 (12.5)
Occupational physical demands, n (%)	Light	566 (26.8)	526 (28.0)	40 (17.2)
	Moderate	984 (46.7)	889 (47.4)	95 (40.8)
	Strenuous	550 (26.1)	454 (24.2)	96 (41.2)
Adequacy of income, n (%)	Quite comfortably off	692 (32.8)	657 (35.0)	35 (15.0)
	Able to manage without much difficulty	780 (37.0)	708 (37.7)	72 (30.9)
	To be careful with money/find it strain to get by from week- to-week	498 (23.6)	392 (20.9)	106 (45.5)
	Prefer not to say	130 (6.2)	113 (6.0)	17 (7.3)
IMD quintiles, n (%)	Q1 – Most affluent	756 (35.9)	681 (36.3)	75 (32.2)
	Q2	463 (22.0)	426 (22.7)	37 (15.9)
	Q3	437 (20.7)	385 (20.5)	52 (22.3)
	Q4	254 (12.0)	227 (12.1)	27 (11.6)
	Q5 – Most deprived	199 (9.4)	157 (8.4)	42 (18.0)
**General health and lifestyle**				
	BMI, mean (SD)	26.9 (5.0)	26.7 (4.8)	29.0 (5.7)
Smoking, n (%)	Never	1,038 (49.2)	933 (49.7)	105 (45.1)
	Ex-/Current	1,061 (50.3)	933 (49.7)	128 (54.9)
Comorbidities (0–11), median (IQR)		2 (1–3)	2 (1–2)	3 (2–3)
Hours/day moving around, n (%)	≥7 hours/day	477 (22.6)	449 (23.9)	28 (12.0)
	5–7 hours/day	547 (25.9)	503 (26.8)	44 (18.9)
	3–5 hours/day	656 (31.1)	583 (31.1)	73 (31.3)
	Less than 3 hour per day	415 (19.7)	330 (17.6)	85 (36.5)
Problems performing usual activities^a^, median(IQR)		1 (1–2)	1 (1–2)	3 (2–3)
Anxiety/depression^b^, median (IQR)		1 (1–2)	1 (1–2)	2 (1–2)
Fit/active as possible by exercising^c^, median (IQR)		2 (1–3)	2 (1–3)	3 (2–4)
**Back pain presentation**				
Back pain group	BP only	1,394 (66.1)	1,304 (69.5)	90 (38.6)
	BP + non-NC leg pain	310 (14.7)	271 (14.5)	39 (16.7)
	BP + NC leg pain	389 (18.4)	286 (15.3)	103 (44.2)
Age of onset	≤ 40 years old	693 (32.9)	624 (33.3)	69 (29.6)
	41–64 years old	782 (37.1)	675 (36.0)	107 (45.9)
	65–74 years old	421 (20.0)	390 (20.8)	31 (13.3)
	75 + years old	190 (9.0)	165 (8.8)	25 (10.7)
BP frequency, n (%)	Rarely/few days	647 (30.7)	624 (33.3)	23 (9.9)
	Some days	586 (27.8)	554 (29.5)	32 (13.7)
	Most days/Every day	849 (40.3)	673 (35.9)	176 (75.5)
BP Troublesome, n (%)	Not at all/Slightly	1,160 (55.0)	1,121 (59.8)	39 (16.7)
	Moderately	646 (30.6)	572 (30.5)	74 (31.8)
	Very or extremely	290 (13.8)	172 (9.2)	118 (50.6)
**Other pain**				
Multisite pain (0–7), median (IQR)		2 (1–3)	2 (1–3)	3 (2–5)
**Age-related adverse health states**				
Frail, n (%)		672 (31.9)	512 (27.3)	160 (68.7)
Fall in the last year, n (%)		712 (33.8)	585 (31.2)	127 (54.5)
Confidence to walk (1–10)^d^, median (IQR)		1 (1–3)	1 (1–2)	7 (2–10)
Mobility decline over the last year, n (%)		596 (28.3)	465 (24.8)	131 (56.2)
Poor sleep quality, n (%)		485 (23.0)	385 (20.5)	100 (42.9)
Urinary incontinence, n (%)		237 (11.2)	184 (9.8)	53 (22.8)
Lack of strength in hands, n (%)		562 (26.7)	436 (23.2)	126 (54.1)
Number of adverse health states, median (IQR)		1 (0–3)	1 (0–3)	4 (2–5)

a. Scored 1–5. 1 = I have no problems doing my usual activities; 5 = I am unable to do my usual activities

b. Scored 1–5. 1 = I am not anxious or depressed; 5 = I am extremely anxious or depressed

c. Scored 1–5. 1 = Strongly agree; 5 = Strongly disagree

d. Higher score represents lower confidence

In Table 3, we present the univariable and multivariable associations between baseline variables and severe pain interference at two-year follow up. The strongest univariate associations were perceived adequacy of income, frailty, time being active, and reporting BP with NC leg pain.

The final model contained the following variables: adequacy of income, number of comorbidities, problems performing usual activities, attitude to exercise, back pain presentation, multisite pain, all the age-related adverse health states and the total number of adverse health states. We identified five baseline variables associated with severe pain interference at 2-year follow up after adjusting for baseline BP troublesomeness. These factors were interference were adequacy of income (careful with money [OR 1.91; 95% CI 1.19–3.06]; prefer not to say [OR 2.22; 95% CI 1.11–4.43]), low endorsement of exercise in later life (OR 1.18; 95% CI 1.02–1.37), neurogenic claudication symptoms (OR 1.68 (95% CI 1.15–2.46)], multisite pain (OR 1.13; 95% CI 1.02–1.24) and low walking confidence (OR 1.15; 95% CI 1.08–1.22).

**Table 3:** Association between independent variables at baseline and severely interfering back pain at two years follow up

Independent variables	Univariable analysis^a^		Multivariable analysis					
			Model 1		Model 2		Model 3	
			**Demographic + General Health + Lifestyle**		Model 1 + Pain		Model 2 + Adverse health states	
	OR (95%CI)	p-value	OR (95%CI)	p-value	OR (95%CI)	p-value	OR (95%CI)	p-value
**Demographic factors**								
Age (years)	1.01 (0.98–1.03)	0.578	-	-	-	-	-	-
Sex (Female vs. Male)	1.12 (0.82–1.53)	0.477	-	-	-	-	-	-
Education								
Secondary	1.15 (0.82–1.60)	0.414	-	-	-	-	-	-
None or primary	1.64 (0.95–2.83)	0.075	-	-	-	-	-	-
Occupational physical demands								
Light	1.00							
Moderate	1.06 (0.70–1.61)	0.769	0.90 (0.59–1.40)	0.659	-	-	-	-
Strenuous	**1.62 (1.06–2.49)**	**0.026**	1.19 (0.75–1.89)	0.448	-	-	-	-
Adequacy of income								
Quite comfortably off	1.00		1.00		1.00		1.00	
Able to manage without much difficulty	1.52 (0.98–2.37)	0.064	1.43 (0.90–2.26)	0.126	1.41 (0.90–2.23)	0.137	1.35 (0.85–2.15)	0.199
To be careful with money/Find it a strain to get by	**2.79 (1.81–4.31)**	**< 0.001**	**2.09 (1.30–3.35)**	**0.002**	**2.10 (1.33–3.31)**	**0.001**	**1.91 (1.19–3.06)**	**0.007**
Prefer not to say	**2.49 (1.29–4.81)**	**0.007**	**2.09 (1.06–4.14)**	**0.034**	**2.36 (1.20–4.64)**	**0.013**	**2.22 (1.11–4.43)**	**0.024**
IMD quintiles								
Q1 – Most affluent	1.00							
Q2	0.75 (0.48–1.17)	0.198	0.70 (0.44–1.11)	0.127	-	-	-	-
Q3	1.13 (0.75–1.71)	0.566	1.16 (0.75–1.78)	0.513	-	-	-	-
Q4	0.94 (0.57–1.56)	0.819	0.80 (0.47–1.37)	0.423	-	-	-	-
Q5 – Most deprived	**1.63 (1.01–2.61)**	**0.044**	1.00 (0.59–1.68)	0.995	-	-	-	-
**General health and lifestyle**								
BMI (Kg/m2)	**1.05 (1.02–1.08)**	**0.001**	1.02 (0.99–1.05)	0.201	-	-	-	-
Smoking (Ex-/current vs. Never)	1.15 (0.85–1.56)	0.354	-	-	-	-	-	-
Comorbidities (0–11)	**1.34 (1.19–1.51)**	**< 0.001**	**1.18 (1.04–1.35)**	**0.012**	**1.14 (1.00-1.30)**	**0.043**	1.09 (0.95–1.25)	0.209
Hours/day moving around								
≥7 hours/day	1.00		1.00					
5–7 hours/day	1.26 (0.75–2.11)	0.383	1.06 (0.63–1.81)	0.820	-	-	-	-
3–5 hours/day	1.48 (0.92–2.39)	0.108	1.07 (0.65–1.77)	0.791	-	-	-	-
Less than 3 hour per day	**2.20 (1.35–3.59)**	**0.002**	1.14 (0.66–1.96)	0.632	-	-	-	-
Problems performing usual activities (1–5)	**1.83 (1.55–2.16)**	**< 0.001**	**1.47 (1.21–1.78)**	**< 0.001**	**1.38 (1.14–1.66)**	**0.001**	1.14 (0.92–1.42)	0.230
Fit/active as possible by exercising (1–5)^b^	**1.47 (1.30–1.67)**	**< 0.001**	**1.28 (1.11–1.48)**	**0.001**	**1.28 (1.12–1.48)**	**< 0.001**	**1.18 (1.02–1.37)**	**0.028**
Anxiety/depression (1–5)	**1.26 (1.05–1.50)**	**0.013**	0.91 (0.74–1.12)	0.372	-	-	-	-
**Back pain presentation**								
Back Pain groups								
BP only	1.00		-	-	1.00		1.00	
BP + non-NC leg pain	1.20 (0.78–1.86)	0.405	-	-	1.08 (0.68–1.71)	0.737	1.08 (0.68–1.72)	0.739
BP + NC leg pain	**2.17 (1.53–3.09)**	**< 0.001**	-	-	**1.70 (1.17–2.46)**	**0.005**	**1.68 (1.15–2.46)**	**0.008**
BP frequency	**1.31 (1.10–1.55)**	**0.002**	-	-	1.10 (1.00-1.45)	0.053	-	-
Age at onset of BP								
≤40 years old	1.00							
41–64 years old	1.30 (0.91–1.86)	0.148	-	-	-	-	-	-
65–74 years old	0.64 (0.40–1.03)	0.066	-	-	-	-	-	-
75 + years old	1.34 (0.78–2.29)	0.293	-	-	-	-	-	-
**Other pain**								
Multisite pain (0–7)	**1.24 (1.14–1.35)**	**< 0.001**	-	-	**1.11 (1.01–1.22)**	**0.026**	**1.13 (1.02–1.24)**	**0.018**
**Age-related adverse health states**								
Frail	**2.71 (1.94–3.78)**	**< 0.001**	-	-	-	-	1.02 (0.66–1.58)	0.922
Fall in the last year	**1.67 (1.23–2.27)**	**< 0.001**	-	-	-	-	1.07 (0.75–1.50)	0.718
Confidence to walk (1–10)^c^	**1.24 (1.18–1.29)**	**< 0.001**	-	-	-	-	**1.15 (1.08–1.22)**	**< 0.001**
Mobility decline over the last year	**1.76 (1.28–2.42)**	**< 0.001**	-	-	-	-	0.81 (0.55–1.18)	0.267
Poor sleep quality	**1.84 (1.34–2.52)**	**< 0.001**	-	-	-	-	1.20 (0.84–1.72)	0.308
Urinary incontinent	**1.52 (1.04–2.24)**	**0.033**	-	-	-	-	0.92 (0.60–1.41)	0.706
Lack of strength in hands	**1.98 (1.44–2.71)**	**< 0.001**	-	-	-	-	0.95 (0.64–1.40)	0.786
Number of adverse health states (0–7)	**1.41 (1.29–1.54)**	**< 0.001**	-	-	-	-	1.11 (0.99–1.25)	0.070

a. Multivariable models were adjusted for baseline back pain troublesomeness

b. Higher score represents less agreement

c. Higher score represents lower confidence

Bold indicated variables that reach statistical significance of p<0.05

## Discussion

A minority of older people with BP will recover over a 2-year period. The majority will stay the same or worsen. Half of this population described their pain as being very or extremely troublesome with a smaller proportion reporting severe pain interference. Five baseline variables were associated with increased risk of reporting severe pain interference at 2 years follow up in the multivariable model after adjusting for baseline BP troublesomeness. These included pain related factors (presenting with BP and NC leg pain and reporting multisite pain), a socioeconomic factor (perceived adequacy of income), an age-related adverse health state (low walking confidence) and a response to a question from the Attitude to Ageing Questionnaire (less agreement with the statement “I keep fit/active as possible by exercising”).

We focused on severe pain interference because it is a potential threat to an older person’s ability to maintain their independence when pain interferes with their daily activities. This approach differs to other studies in this area who have used different ways to define a poor outcome. Definitions of persistent back pain include a report of pain scored ≥ 1 on numerical rating scale (NRS) of 0–10 [14] or a back pain NRS of 3/10 or higher at both six and 12 months [11, 12]. The presence of persistent disability has been defined as a Roland and Morris Disability questionnaire score of 4/24 or higher at both six and 12 months [11, 12] or by the presence of restricting back pain assessed during monthly interviews with a report of staying in bed or cutting down on usual activities due back pain [15]. Other studied used the RMDQ [4, 38] or Brief Pain Inventory [38] as a continue measure. Despite difference in outcomes, there were commonalities with previous studies. A report of multi-site pain was associated with poor outcome in our cohort. Similar pain related factors including widespread pain [12] and musculoskeletal comorbidities [11] in particular, hip and knee osteoarthritis [38] have been identified previously as associated with BP outcomes in older people. A presentation of back pain with NC leg pain has a substantial impact on an older person. In this study, it increased the odds of a poor outcome by around 70%. Reports of back and leg pain and a diagnosis of spinal stenosis have been associated with poor outcomes in a previous longitudinal study [12]. Our findings add further evidence that pain presentations (NC leg pain) and multisite pain maybe used to identify older people who are risk of poor BP outcomes.

Perceived inadequacy of income was also identified as risk factor for severe pain interference. We are not aware of any other studies that have investigated this as a prognostic factor for older people with BP. In addition, those participants who answered the question about income with “prefer not to say” were twice as likely to report severe pain interference at two years compared to those who were quite comfortably off. To understand this relationship, we looked at the demographic characteristics by adequacy of income. Among participants who responded “prefer not to say”, a greater proportion lived in more deprived areas, rated the physical demands of their main occupation as strenuous/very strenuous and reported no or primary education only (data not shown) compared to the other groups. This suggests participants who responded with “prefer not to say” faced financial challenges which they preferred not to reveal. The link between social determinants of health (which includes factors related to income/wealth, economic stability, education and employment) and low back pain outcomes was investigated in a systematic literature review who reported that low education, low income and low socioeconomic status were consistently associated with poor low BP outcomes [39]. Despite the biopsychosocial model of pain being developed over 30 years ago [40], the social aspect of this model receives little attention compared to the biological and psychological aspects. There is evidence that people from lower socioeconomic backgrounds are less likely to access BP treatments [41] which may contribute to poorer outcomes but a better understanding is needed of how socioeconomic factors contribute to the persistence of BP. The two remaining risk factors (low walking confidence and attitude to exercise) have not been studied previously in this population in regard to BP outcomes as far as we are aware.

These risk factors were identified using questions that could easily be asked during a clinical consultation making the findings easily transferable to clinical practice to identify older people who are at risk of poor outcomes with the aim of intervening. However, further research is needed before these can be applied to clinical practice. Further validation of these findings in different research settings or countries is required to apply these findings more broadly. We do not know if identifying older people at risk of poor outcome based on these factors and intervening improves outcomes. Care should be taken not to conflate prediction with causation [42]. Although, we studied factors that are also potential treatment targets (e.g. beliefs about exercise), this study does not confirm their role in the development of persistent disabling pain in this population. This would require further evaluation through interventional trials.

A limitation of this study is that we relied on self-reported measures rather than radiological confirmation of a back pain diagnosis. This may have resulted in some participants being misclassified, as BP may exist alongside other conditions such as vascular claudication which have a similar symptom presentation to NC. It also relied on participants’ recall of events such as falls and their perceptions around their walking ability, which may not reflect their true walking ability. We also do not know if participants sought treatments for their back and leg symptoms which may have influenced their recovery trajectory.

## Conclusion

After adjusting for baseline pain severity, we identified five factors that were associated severe pain limitation at two years follow up among a cohort of community dwelling older people reporting back and leg pain. These included other pain characteristics, walking confidence and attitude to exercise. We also identified a socioeconomic factor (perceived adequacy of income). Future research should focus on whether identifying individuals using the identified risk factors in order to intervene improves back pain outcomes for older people.

## Electronic Supplementary Material

Below is the link to the electronic supplementary material.

Supplementary Material 1

## Data Availability

The datasets analysed during the current study are available from the Chief Investigator (Professor Sallie Lamb, s.e.lamb@exeter.ac.uk) on reasonable request.

## Abbreviations

BP: Back pain
NC: Neurogenic claudication
