# Supplementary material for: Predicting persistent back pain causing severe interference with daily activities among community-dwelling older adults: the OPAL cohort study
Source: BMC Geriatr. 2024 Nov 14;24:942. doi: 10.1186/s12877-024-05504-1 (PMC11566404; doi:10.1186/s12877-024-05504-1)
Supplement: Supplementary file 1 — Supplementary Material 1 [file 12877_2024_5504_MOESM1_ESM.pdf]

Supplementary materials

Questions completed by participants

Outcome – severe back pain interference

In the **PAST 6 WEEKS**, how much has your back pain interfered with your daily activities on a scale of 0 to 10, where 0 = no interference and 10 = unable to carry out any activities at all?

Independent variables (baseline factors):

Demographics

Age: taken from date of birth on baseline form.

Sex: Male/Female.

Education:
What is the highest level of education you have completed?
- None or primary education
- Secondary education
- Higher professional or university education

Physical demands of occupation:
How physically demanding was your **MAIN** occupation?
- Very light
- Light
- Moderate
- Strenuous
- Very strenuous

Deprivation: participants were allocated an Index of multiple deprivation score (IMD (0-100 score) based on their postcode at baseline.

Adequacy of income:
Thinking about the cost of living as it affects you, which of the following best describes your situation? Please cross (X) **ONE** box.
- Find it a strain to get by from week-to-week
- Have to be careful with money
- Able to manage without much difficulty
- Quite comfortably off
- Prefer not to say

General health

Body Mass Index (BMI): calculated using self-reported height and weight at baseline.

Comorbidities:
Has your doctor or nurse ever told you that you have any of the following health conditions? **IF YES**, please place a cross (X) next to **ALL** that apply.
- Arthritis
- Angina or heart troubles
- Cancer (active)
- Chronic lung disease
- Dementia
- Diabetes (Types I or II)
- Digestive problems
- High blood pressure

Osteoporosis
Parkinson's disease
Peripheral Vascular Disease
Stroke
Other, please state

Anxiety and depression:
Please cross (X) **ONE** box that best describes your health **TODAY**.
Anxiety/depression
I am not anxious or depressed
I am slightly anxious or depressed
I am moderately anxious or depressed
I am severely anxious or depressed
I am extremely anxious or depressed

Lifestyle
Smoking:
Which of the following best describes your **current** cigarette smoking status?
Please cross (X) **ONE** box.
Never smoked
Former smoker
Current smoker

Physical activity:
In the **PAST WEEK** how many hours a day have you typically spent **moving around on your feet?** This includes work-related activities, household work (like cleaning), garden work (like mowing / raking the lawn), walking (from place to place or for leisure / sports), and running at a slow or fast pace. Please cross (X) **ONE** box.
Less than 1 hour per day
1 to less than 3 hours per day
3 to less than 5 hours per day
5 to less than 7 hours per day
7 hours or more per day

Attitude to exercise:
Please cross (X) **ONE** box that best describes how you feel about each statement.
I keep as fit and active as possibly by exercising (1-5 scale)
Strongly agree = 1
Neither agree or disagree = 3
Strongly disagree = 5

Function:
Please cross (X) **ONE** box that best describes your health **TODAY**.
Usual activities (e.g. work, study, housework, family or leisure activities)
I have no problems doing my usual activities
I have slight problems doing my usual activities
I have moderate problems doing my usual activities
I have severe problems doing my usual activities
I am unable to do my usual activities

Pain
Report of back and leg pain:
Are you troubled by back pain or related symptoms?
No/Yes

Frequency:
IF YES, how often have you had back pain or related symptoms in the **LAST 6 WEEKS**? Please cross (X) **ONE** box.

- Every day
- Most days
- Some days
- Few days
- Rarely

Troublesomeness:
How troublesome has your back or related symptoms been during the **PAST 6 WEEKS**? Please cross (X) **ONE** box.

- Not at all troublesome
- Slightly troublesome
- Moderately troublesome
- Very troublesome
- Extremely troublesome

Spread of symptoms:
In the **PAST 6 WEEKS**, have you had back pain or other symptoms such as tingling, numbness or heaviness that travelled from your back into your buttocks or legs?

No/Yes

IF YES, please answer the questions below:
Have these symptoms spread below the knee?

Yes/No

Does standing make the pain or symptoms in your buttocks or legs **worse**?

Yes/No

Does walking make the pain or symptoms in your buttocks or legs **worse**?

Yes/No

Does sitting down make the pain or symptoms in your buttocks or legs **better**?

Yes/No

Does bending forward (for example to push a shopping trolley) make the pain or symptoms in your buttocks or legs **better?**

Yes/No

Multisite pain:
Have you at any time during the **LAST 6 WEEKS** had trouble (ache, pain, discomfort) in the following areas of your body? **IF YES,** please place a cross (X) in the box next to **ALL** that apply.

- Neck
- Shoulders
- Elbows
- Wrist/hands
- Upper back
- Low back (small of the back)
- One or both hips/thighs
- One or both knees
- One or both ankles/feet

Age-related adverse health states

Frailty:

Do you feel physically healthy?

Yes/No

Have you lost a lot of weight recently without wishing to do so? A lot is 6kg (13 pounds) or more during the last 6 months or 3kg (7 pounds) or more in the last month.

Yes/No

Do you experience problems in your daily life due to difficulty in walking?

Yes/No

Do you experience problems in your daily life due to difficulty maintaining your balance?

Yes/No

Do you experience problems in your daily life due to poor hearing?

Yes/No

Do you experience problems in your daily life due to poor vision?

Yes/No

Do you experience problems in your daily life due to lack of strength in your hands?

Yes/No

Do you experience problems in your daily life due to physical tiredness?

Yes/No

Are you able to cope with problems well?

Yes/No

Do you live alone?

Yes/No

Do you receive enough support from other people?

Yes/No

Do you have problems with your memory?

Sometimes/Yes/No

Do you sometimes miss having other people around you?

Sometimes/Yes/No

Have you felt down during the last month?

Sometimes/Yes/No

Have you felt nervous or anxious during the last month?

Sometimes/Yes/No

Mobility decline:

Compared to **one year ago**, how would you rate your walking in general **NOW**? Please cross (X) **ONE** box

Much better now than one year ago
Somewhat better than one year ago
About the same
Somewhat worse than one year ago
Much worse now than one year ago

Walking self-efficacy:

How much confidence do you have that you would be able to safely walk a long distance such as ½ a mile? (1-10 scale, 1=no confidence to 10=complete confidence)

Falls:

In the last **12 MONTHS**, have you had any fall including a slip or trip following which you have come to rest on the ground, floor or lower level? Please cross (X) **ONE** box.

I have not fallen in the last year
I have fallen once in the last year
I have fallen more than once in the last year

Incontinence:

Are you incontinent of urine? Please cross (X) **ONE** box.

- Never
- Less than once per week
- Less than once per day
- More often
- Or do you have a catheter managed for you?

Sleep:

During the **PAST MONTH** how would you rate your sleep quality overall? Please cross (X) **ONE** box.

- Very good
- Fairly good
- Fairly bad
- Very bad

Grip strength:

Do you experience problems in your daily life due to lack of strength in your hands?

- Yes/No

Supplementary Data. Missing Data and Multiple Imputation

Multiple imputation procedure

Missing data is an important concern for longitudinal studies, especially those on older adults. It has been argued that the imputation of missing covariates data in medical research is always better than the complete case (1). Excluding subjects with missing values leads to a reduction in the sample study size, may result in incorrect estimates of specific predictors, and may diminish the predictive power of the working statistical model.

In order to reduce potential biases arising from missing data, multiple imputations were used. MICE package in Stata was performed with 100 iterations to create 20 imputed datasets using the 25 independent baseline variables plus 5 back pain-related baseline variables plus outcome. Imputation methods were continuous variables: predictive mean matching (pmm) or linear regression (regress); 2-level factors: binary logistic regression (logit); and >2-level factors: multinomial regression (mlogit) (ordered or unordered) (see Table S1 below).

Table S1. Missing data for each independent baseline variable

**Baseline factors**	**Missing data, n (%)**	**Imputation method**
**Demographic**		
Age	0 (0.0)	-
Sex	0 (0.0)	-
Education	12 (0.6)	mlogit
Occupational physical demands	9 (0.4)	mlogit
Adequacy of income	9 (0.4)	mlogit
IMD	0 (0.0)	-
**General health and lifestyle**		
BMI	76 (3.6)	regress
Smoking	10 (0.5)	logit
Comorbidities	0 (0.0)	-
Hours/day moving around	14 (0.7)	mlogit
Problems performing usual activities	7 (0.3)	pmm
Anxiety/depression	20 (1.0)	pmm
Fit/active as possible by exercising	10 (0.5)	pmm
**Back pain presentation**		
Back pain group	16 (0.8)	mlogit
Age of onset	23 (1.1)	mlogit
BP frequency	27 (1.3)	pmm
BP Troublesome	13 (0.6)	pmm
**Other pain**		
Multisite pain	0 (0.0)	-
**Age-related adverse health states**		
Frail	23 (1.1)	logit
Fall in the last year	8 (0.4)	logit
Confidence to walk	4 (0.2)	pmm
Mobility decline over the last year	8 (0.4)	logit
Poor sleep quality	8 (0.4)	logit
Urinary incontinence	9 (0.4)	logit
Lack of strength in hands	10 (0.5)	logit

Table S2. Comparison of baseline characteristics between original OPAL cohort sample and analytic sample

Baseline factors		OPAL baseline cohort	Analytic sample
		N=5,409	N=2,109
**Demographic**			
Age (years), mean (SD)		74.9 (6.8)	74.2 (6.3)
Sex, n (%)	Female	2,783 (51.5)	1,170 (55.5)
Education, n (%)	Higher education	1,912 (35.4)	807 (38.3)
	Secondary	3,051 (56.4)	1,152 (54.6)
	None or primary	408 (7.5)	138 (6.5)
Occupational physical demands, n (%)	Light	1,446 (27.0)	566 (26.8)
	Moderate	2,533 (47.2)	984 (46.7)
	Strenuous	1,385 (25.82)	550 (26.1)
Adequacy of income, n (%)	Quite comfortably off	1,763 (32.6)	692 (32.8)
	Able to manage without much difficulty	2,035 (37.6)	780 (37.0)
	To be careful with money/find it strain to get by	1,225 (22.7)	498 (23.6)
	Prefer not to say	348 (6.4)	130 (6.2)
IMD quintiles, n (%)	Q1 – Most affluent	1,844 (34.1)	756 (35.9)
	Q2	1,166 (21.6)	463 (22.0)
	Q3	1,155 (21.4)	437 (20.7)
	Q4	662 (12.2)	254 (12.0)
	Q5 – Most deprived	582 (10.8)	199 (9.4)
**General health and lifestyle**			
	BMI, mean (SD)	26.6 (4.8)	26.9 (5.0)
Smoking, n (%)	Never	2,689 (49.7)	1,038 (49.2)
	Ex-/Current	2,704 (50.0)	1,061 (50.3)
Comorbidities (0-11), median (IQR)		2 (1-2)	2 (1-3)
Hours/day moving around, n (%)	≥7 hours/day	1,201 (22.2)	477 (22.6)
	5-7 hours/day	1,442 (26.7)	547 (25.9)
	3-5 hours/day	1,627 (30.1)	656 (31.1)
	Less than 3 hour per day	1,099 (20.3)	415 (19.7)
Problems performing usual activities[a], median(IQR)		1 (1-2)	1 (1-2)
Anxiety/depression[b], median (IQR)		1 (1-2)	1 (1-2)
Fit/active as possible by exercising[c], median (IQR)		2 (1-3)	2 (1-3)
**Age-related adverse health states**			
Frail, n (%)		1,465 (27.1)	672 (31.9)
Fall in the last year, n (%)		1,569 (29.0)	712 (33.8)
Confidence to walk (1-10)[d], median (IQR)		1 (1-3)	1 (1-3)
Mobility decline over the last year, n (%)		1,375 (25.4)	596 (28.3)
Poor sleep quality, n (%)		1,053 (19.5)	485 (23.0)
Urinary incontinence, n (%)		547 (10.1)	237 (11.2)
Lack of strength in hands, n (%)		1,236 (22.9)	562 (26.7)
Number of adverse health states, median (IQR)		1 (0-3)	1 (0-3)

a. Scored 1-5. 1=I have no problems doing my usual activities; 5= I am unable to do my usual activities
b. Scored 1-5. 1=I am not anxious or depressed; 5=I am extremely anxious or depressed
c. Scored 1-5. 1=Strongly agree; 5=Strongly disagree
d. Higher score represents lower confidence

Reference

1. Janssen KJ, Donders AR, Harrell FE, Jr., Vergouwe Y, Chen Q, Grobbee DE, et al. Missing covariate data in medical research: to impute is better than to ignore. J Clin Epidemiol. 2010;63(7):721-7.
